# Food addiction in behavioral addictions: a network approach

**DOI:** 10.3389/fpsyg.2026.1703292

**Published:** 2026-02-25

**Authors:** Anahí Gaspar-Pérez, Roser Granero, Fernando Fernández-Aranda, Magda Rosinska, Noelia Sabariegos-Campos, Cristina Artero, Silvia Ruiz-Torras, Ashley N. Gearhardt, Zsolt Demetrovics, Andrea Czakó, Joan Guàrdia-Olmos, Susana Jiménez-Murcia

**Affiliations:** 1 Doctoral Program in Clinical and Health Psychology, University of Barcelona, Barcelona, Spain; 2 Department of Clinical Psychology, University Hospital of Bellvitge-IDIBELL, Barcelona, Spain; 3 Psychoneurobiology of Eating and Addictive Behaviors Group, Neuroscience Program, Bellvitge Biomedical Research Institute (IDIBELL), Barcelona, Spain; 4 Ciber Fisiopatología Obesidad y Nutrición (CIBERobn), Instituto de Salud Carlos III, Madrid, Spain; 5 Department of Psychobiology and Methodology, Autonomous University of Barcelona, Barcelona, Spain; 6 Department of Clinical Sciences, School of Medicine and Health Sciences, University of Barcelona, Barcelona, Spain; 7 Centre de Serveis de Psicologia, Fundació Josep Finestres, Universitat de Barcelona, Barcelona, Spain; 8 Facultat de Psicologia, Secció de Psicologia Quantitativa, Universitat de Barcelona, Barcelona, Spain; 9 UB Institute of Complex Systems, Universitat de Barcelona, Barcelona, Spain; 10 Institute of Neuroscience, Universitat de Barcelona, Barcelona, Spain; 11 Department of Psychology, University of Michigan, Ann Arbor, MI, United States; 12 Flinders University Institute for Mental Health and Wellbeing, College of Education, Psychology and Social Work, Flinders University, Bedford Park, SA, Australia; 13 Institute of Psychology, ELTE Eötvös Loránd University, Budapest, Hungary; 14 Center of Excellence in Responsible Gaming, University of Gibraltar, Gibraltar

**Keywords:** addictive behaviors, behavioral addictions, clinical profile, food addiction, network analysis

## Abstract

**Background/aims:**

The comorbidity of mental disorders is a well-documented phenomenon. However, the co-occurrence of and underlying mechanisms of food addiction (FA) and behavioral addictions (BAs) have been scarcely investigated. Therefore, the aims of this study were as follows: (a) to perform a network analysis to explore the interrelationships between the clinical profile of patients seeking treatment for gaming disorder, compulsive buying–shopping disorder (CBSD), compulsive sexual behavior disorder, and the comorbid presence of multiple BAs and FA; (b) to identify the core symptoms (central nodes) and correlates contributing to the clinical profile of patients with this comorbidity; and (c) to determine empirically derived clusters of nodes depending on the presence or absence of FA.

**Methods:**

A sample of 209 participants (64.6% men and 35.4% women) was assessed using a semi-structured clinical interview to diagnose BAs, along with self-reported psychometric assessments of FA, general psychopathology, personality traits, emotion regulation, and impulsivity. Separate networks were estimated according to the presence or absence of FA.

**Results:**

The nodes with the highest centrality indices among patients with FA + were self-directedness, followed by global psychopathological distress, age, and harm avoidance. Self-directedness was also identified as the most relevant bridging node among patients with FA+. The number of communities (clusters of nodes) and their composition varied depending on the presence of comorbid FA.

**Conclusion:**

The profile observed in patients with both BAs and FA appears more complex than that observed in those without FA, and this increased complexity may influence the course of the disorders as well as treatment outcomes.

## 1 Introduction

Epidemiological research estimates that 31.1% of adults experience concurrent psychiatric disorders, which are associated with greater functional impairment and poorer treatment outcomes (Forman–Hoffman et al., 2018; Ziobrowski et al., 2021). In this context, the co-occurrence of food addiction (FA) with other mental health conditions, such as gambling disorder and substance use disorders (SUDs), has received increasing attention in recent years (Jiménez-Murcia et al., 2017a; Etxandi et al., 2021; Miranda-Olivos et al., 2022). This growing interest highlights the need to better understand the interplay of transdiagnostic factors, including personality traits (Watson and Clark, 2023), which have been consistently linked to the development, severity, and course of mental disorders, as well as to treatment outcomes (Santesteban-Echarri et al., 2021). Notably, high neuroticism and low levels of conscientiousness, extraversion, and openness are common across mood, anxiety, and psychotic disorders (Marshall et al., 2012; Dupuis et al., 2016; Ohi et al., 2016). Moreover, high neuroticism has been associated with an increased risk of adjustment, personality, alcohol, and SUDs, while psychoticism may contribute to heightened susceptibility to stress, maladaptive emotional regulation, and more severe psychopathological symptoms (Christensen et al., 2021).

### 1.1 Understanding food addiction

FA is characterized by unregulated and excessive consumption of highly palatable foods (such as ultra-processed foods and other foods rich in fat, sugar, and salt), in a quantity disproportionate to homeostatic energy requirements (Imperatori et al., 2016). The definition of FA as a diagnostic entity is controversial, but this study has identified behavioral and biological markers explaining the key symptoms of addiction, such as tolerance, withdrawal, larger amounts consumed than intended, lack of capacity to cut down, or continued eating despite knowledge of the negative consequences (Gearhardt et al., 2009; Penzenstadler et al., 2019). Although FA is not currently recognized as a clinical disorder in diagnostic taxonomies, addictive-like eating of hyperpalatable foods has been associated with psychoneurological deficits and functional impairment (LaFata et al., 2024).

The prevalence of FA in the general adult population is estimated to be approximately 14–20%, and approximately 12–15% in the young population (LaFata and Gearhardt, 2022; LaFata et al., 2024). Higher prevalences of FA have been estimated within middle-aged women (45–65 years) and among people with overweight and obesity (Imperatori et al., 2016; Burrows et al., 2018). Studies have related FA to high body mass index (BMI), poor sleep quality, depression or anxiety symptoms, and sedentary lifestyles (Camacho-Barcia et al., 2021; Romero-Blanco et al., 2021; Florio et al., 2022). Etiological research has also shown that FA and SUD share parallel mechanisms, including behavioral patterns (e.g., loss of control, persistence of the behavior despite negative consequences, abandonment of other activities, and excessive time devoted to the behavior, among others) and neurobiological processes (e.g., shared pathways in the opioid, cannabinoid, and dopaminergic systems, as well as effects on reward circuits and neuronal areas related to inhibitory control) (Imperatori et al., 2016; Gupta et al., 2020; Gearhardt and Schulte, 2021; Florio et al., 2022; Unwin et al., 2025).

In addition, FA has shown comorbidities with other mental disorders, especially anxiety and mood disorders (Burrows et al., 2018; Oliveira et al., 2020). Meule and Gearhardt’s systematic review (2019) reported an association between FA and post-traumatic stress disorder (PTSD), attention deficit/hyperactivity disorder (ADHD), suicidal tendencies, non-suicidal self-injury (NSSI), difficulty in emotional regulation, and poor sleep quality (Meule and Gearhardt, 2019). FA has also been linked to eating disorders (EDs), particularly binge eating disorder (BED) and bulimia nervosa (BN), in features such as impulsivity and high levels of psychopathology (elevated emotional dysregulation and negative moods) (Munguía et al., 2022a, 2022b).

### 1.2 Behavioral addictions: definition and scope

When exploring behavioral addictions (BAs), these conditions are also characterized by functional impairment across multiple areas of an individual’s life (financial, familial, personal, occupational or educational, social, physical, or psychological), resulting from recurrent difficulties in controlling impulses to engage in a specific behavior despite negative consequences (Mei et al., 2024). Several studies have observed the presence of shared components among BAs, encompassing personality traits and cognitive profiles, as well as genetic determinants, neurobiological mechanisms, and treatment-response patterns (Kim and Hodgins, 2018; Goslar et al., 2020; Kotyuk et al., 2020). The most prevalent BAs reported in adult populations are gambling disorder (1.41%), compulsive buying–shopping disorder (CBSD) (5%), and gaming disorder (3.05%) (Black et al., 2010; Maraz et al., 2016; Stevens et al., 2021; Tran et al., 2024). Prevalence estimates for compulsive sexual behavior disorder in adults have been under-researched and under-determined, but a recent systematic review identified prevalence rates ranging from 3.3 to 40.3% in clinical samples (Kürbitz and Briken, 2021a). BAs present comorbidity with other mental disorders, mainly with anxiety disorders, depression, ADHD, social phobia, PTSD, obsessive-compulsive disorder (OCD), SUDs, and some EDs, such as BN and BED (Müller et al., 2019, 2023; Granero et al., 2021a; Kürbitz and Briken, 2021a; Grant Weinandy et al., 2023). It has also been proposed that the variety of BAs show similar clinical patterns both in comparison to control groups and as a function of the severity of addictive behaviors (Li et al., 2025).

To date, research on the comorbidity between FA and BAs has primarily focused on gambling disorder, with studies reporting a prevalence of FA of approximately 9.2% (Jiménez-Murcia et al., 2017a; Etxandi et al., 2021). However, this relationship has been scarcely explored in other types of BAs. The study by Müller et al. (2018) examined the relationship between FA and different BAs among bariatric surgery patients, and the results showed an association between FA and CBSD and internet use disorder (Müller et al., 2018). Another study on BAs reported an FA prevalence of 22.49% in a sample including individuals with gaming disorder, CBSD, and compulsive sexual behavior disorder (Gaspar-Pérez et al., 2025). In that study, individuals with comorbid FA exhibited higher emotional distress and impulsivity levels, as well as greater difficulties in daily functioning. However, little is known about the complex interrelationships among variables underlying the comorbidity between FA and different BAs.

### 1.3 Network approach to comorbidity

Network analysis has traditionally been regarded as a data-analytic tool, but its application in the health sciences has highlighted its potential to reinterpret clinical phenomena. By modeling symptoms and related factors as interconnected nodes, network analysis allows researchers and clinicians to move beyond a purely descriptive approach, offering insights into the dynamic structure, interdependencies, and potential causal pathways underlying complex mental health conditions. In psychiatry, the network approach posits that mental disorders emerge from dynamic interactions among symptoms, which are interconnected through biological, psychological, and social mechanisms. Strong associations can generate feedback loops that render symptoms self-sustaining, with the activation of one symptom facilitating the activation of others (McNally, 2016a; Borsboom, 2017a). Disorders emerge when symptom networks remain active over time, while recovery involves reducing symptom intensity, weakening interconnections, or both (McNally, 2016a). Although symptom associations do not imply causality, identifying central symptoms within the network offers a clinically relevant strategy, as modifying these nodes may influence other interconnected symptoms and guide targeted interventions (Borsboom, 2017a; Pan et al., 2024).

Network analysis has been used in different fields of psychology and psychiatry to investigate disorders such as depression, PTSD, OCD, personality disorders, and SUD, among others (McNally, 2016a; Borsboom, 2017a; Epskamp et al., 2018a). In BAs, this type of analysis has been performed for gambling disorder (Mestre-Bach et al., 2022; Oh et al., 2023; Somma et al., 2023; Błoch and Misiak, 2024; Granero et al., 2024; Lucas et al., 2024), problematic internet use (PIU) (Lu et al., 2022, 2023; Sit et al., 2023; Jeong et al., 2024; Sun et al., 2024; Yu et al., 2024; Feng et al., 2025), gaming disorder (Yuan et al., 2022; Sit et al., 2023), problematic smartphone use (PSU) (Guo et al., 2022; Kabadayi, 2024; Li et al., 2024; Tang et al., 2024), and compulsive sexual behavior disorders (Navandar et al., 2021; Marchetti, 2023; Scoglio et al., 2025; Xu et al., 2025). The network approach has also been employed for the study of FA, specifically in comorbid studies exploring the association of this construct with BED and obesity (Carbone et al., 2023) and assessing their links with emotion regulation, impulsiveness, and quality of life (Dejavitte et al., 2025a). These studies found that, in obese patients, FA is closely associated with the presence of depressive symptoms. In contrast, in individuals with BED, it is disordered eating behaviors such as binge eating and frequent grazing that explain the relationship with FA. Furthermore, these studies identified impulsivity as the central symptom in the relationship between FA and emotional dysfunction (Carbone et al., 2023; Dejavitte et al., 2025b). This suggests that the presence of FA constitutes a differential feature in the psychopathology of different clinical populations. However, to date, no studies employing network analysis have examined the relationships between FA and various BAs, representing a significant gap in the literature.

### 1.4 Justification and objectives of the study

In summary, due to the need for empirical research analyzing the comorbidity between FA and different BAs and the advantages of employing network analysis for visualizing the complex underlying mechanisms, the aims of this study are as follows: (a) to perform a network analysis to explore the interrelationships between the clinical profile (including core BA symptoms, psychological distress, impulsivity, and personality dimensions) of patients seeking treatment for gaming disorder, CBSD, or compulsive sexual behavior disorder, and the comorbid presence of multiple BAs and FA; (b) to identify the core symptoms (central nodes) and correlates contributing to the clinical profile of patients with this comorbidity; and (c) to determine empirical clusters of nodes depending on the presence or absence of FA.

To our knowledge, this is the first study to use a network approach to examine the dual presence of FA and different BAs. We believe that this study, in addition to enhancing the understanding of the underlying mechanisms linking FA and BAs, may contribute to the identification of clinically relevant implications for the therapeutic management of both conditions. As suggested by the scientific literature and clinical practice, identifying core symptoms is essential, as these may serve as strategic targets in the design and implementation of effective therapeutic interventions (Borsboom, 2017a). We hypothesize that the network structure will be different for patients with FA (in terms of psychopathological status and personality profile) compared with patients without FA. In addition, this study could provide transdiagnostic insight into the interactions between the symptoms that occur in the comorbidity of these clinical conditions.

## 2 Materials and methods

### 2.1 Participants and procedure

This study included an initial sample of 209 participants who were seeking treatment from the Behavioral Addictions Unit of the Department of Clinical Psychology of the University Hospital of Bellvitge (Barcelona, Spain) between June 2016 and December 2023. Patients who were over 18 years old and requested treatment for gaming disorder, CBSD, or compulsive sexual behavior disorder were included. Exclusion criteria included the presence of any current diagnosis of an ED or any other addiction, according to the Diagnostic and Statistical Manual of Mental Disorders (DSM-5) (American Psychiatric Association, 2013). The patients were classified according to the presence or absence of FA.

Assessments were administered under routine clinical supervision, with clinicians ensuring full completion of all instruments and providing clarification when necessary; consequently, no participants were excluded due to missing data. The same assessment instruments and diagnostic criteria were consistently applied throughout the entire recruitment period.

### 2.2 Assessment

#### 2.2.1 Semi-structured clinical interview

Psychologists and psychiatrists with expertise in the field of BAs and EDs performed the diagnosis of all BAs through a face-to-face semi-structured clinical interview. In addition to the clinical variables, sociodemographic variables were evaluated, including sex, marital status, educational level, employment status, and the social position index (calculated according to the Hollingshead scale, which generates a classification based on marital status, educational level, work situation, and professional standing) (Hollingshead, 2011).

#### 2.2.2 Diagnostic status

##### 2.2.2.1 Gaming disorder

It was measured based on the DSM-5 criteria (American Psychiatric Association, 2013; Petry et al., 2014), considering a 12-month period to establish the diagnosis (Deleuze et al., 2017; Billieux et al., 2019).

##### 2.2.2.2 Compulsive buying–shopping disorder

It was assessed following the guidelines established by McElroy et al. (1994), which are widely accepted by the scientific community (McElroy et al., 1994; Tavares et al., 2008).

##### 2.2.2.3 Compulsive sexual behavior disorder

A self-reported list of items was used to assess this clinical condition, based on the DSM-IV-TR (American Psychiatric Association, 2000), specifically in the Sexual Disorders Not Otherwise Specified section (302.9). These items are fully aligned with the criteria proposed in the International Classification of Diseases, 11th edition (ICD-11), as “Compulsive Sexual Behavior Disorder,” classified under impulse control disorders (ICDs) (World Health Organization, 2022).

#### 2.2.3 Self-report measures

All participants individually completed self-report questionnaires under the supervision of an experienced psychologist (see Supplementary material for internal consistency of the study questionnaires).

##### 2.2.3.1 The Yale Food Addiction Scale 2.0 (YFAS-2)

The Yale Food Addiction Scale 2.0 (YFAS-2) (Gearhardt et al., 2016) is a self-report questionnaire adapted from the DSM-5 criteria for SUD to determine addictive eating behaviors. It consists of 35 items with an eight-point Likert-type scale assessing 11 symptoms. This questionnaire establishes severity ranging from mild (2 to 3 symptoms), moderate (4 to 5 symptoms), to severe (6 to 11 symptoms). The Spanish validation of the YFAS-2 (Granero et al., 2018) was used in this study, which reported an internal consistency of 0.94 (*α* coefficient). The threshold for a positive screening result was established according to specific criteria determined in previous psychometric validation studies. This criterion involved the presence of at least two symptoms, accompanied by a clinically significant level of functional impairment or psychological distress. The internal consistency of the total score for this study was *α* = 0.90.

##### 2.2.3.2 Pathological buying screener

Pathological buying screener (PBS) (Müller et al., 2015) is a self-report scale used to assess compulsive buying behavior, has a total of 13 items, and was translated from English into Spanish (Fernández-Aranda et al., 2019) following the translation and adaptation guidelines of the International Test Commission (International Test Commission, 2017). The questionnaire showed good internal consistency (α = 0.86). Cronbach’s alpha for Time 1 was 0.85 and for Time 2 was 0.84.

##### 2.2.3.3 Impulsive behavior scale

Impulsive Behavior Scale (UPPS-P) (Whiteside and Lynam, 2001) measures five facets of impulsive behavior using five items: negative urgency (NU), lack of perseverance (LP), lack of premeditation (LPM), sensation seeking (SS), and positive urgency (PU). This study used the Spanish version of the UPPS-P (Verdejo-García et al., 2010), which obtained good reliability (Cronbach’s α between 0.79 and 0.93) and external validity. The total score of this scale showed an internal consistency in our sample of α = 0.90.

##### 2.2.3.4 Difficulties in emotion regulation strategies

Difficulties in emotion regulation strategies (DERS) (Gratz and Roemer, 2008) assesses emotional dysregulation by means of 36 items divided into six subscales: non-acceptance of emotional responses, difficulties engaging in goal-directed behavior when having strong emotions, lack of emotional awareness, impulse-control difficulties, limited access to emotional regulation strategies, and lack of emotional clarity. Higher scores indicate greater problems with emotion regulation. This instrument has been validated in a Spanish population (Wolz et al., 2015), which was used for our study, obtaining an internal consistency of α = 0.93 for the total score in our sample.

##### 2.2.3.5 Temperament and character inventory—revised

Temperament and Character Inventory—Revised (TCI-R) (Cloninger, 1999) includes 240 items divided into seven personality dimensions: four associated with temperament (harm avoidance, novelty seeking, reward dependence, and persistence) and three with character (cooperation, self-directedness, and self-transcendence). We used the Spanish version (Gutiérrez-Zotes et al., 2004) for our sample, with a reliability in the seven dimensions ranging between 0.77 and 0.84.

##### 2.2.3.6 Symptom checklist—revised

Symptom Checklist—Revised (SCL-90-R) (Derogatis, 1990) is a questionnaire used to measure various psychological and psychopathological symptoms by means of 90 items grouped into nine primary symptom dimensions: somatization, obsession–compulsion, interpersonal sensitivity, depression, anxiety, hostility, phobic anxiety, paranoid ideation, and psychoticism. Additionally, this test yields the following: (a) a global severity index (GSI), (b) a positive symptom distress index (PSDI), and (c) a positive symptom total (PST). Validation in the Spanish population (Derogatis, 2002) of this questionnaire has given good reliability of the scales ranging between 0.81 and 0.90, with a retest reliability ranging between 0.78 and 0.90. Our sample obtained an internal consistency for the total instrument score of α = 0.98.

### 2.3 Ethics

This study was conducted in accordance with the latest version of the Declaration of Helsinki. The University Hospital Clinical Research Ethics Committee approved the study (Refs. 19/21 of 4/11/21), and written informed consent was obtained from all participants.

### 2.4 Statistical analysis

The data analysis was conducted using Stata 18 for Windows (StataCorp, 2023) for the comparison between the groups. Chi-square (*χ*^2^) tests were employed for categorical variables and t-tests for quantitative measures. The effect sizes were measured using the standardized coefficient Cramer’s *V* for contingency tables (interpreted as mild to moderate to large effects for values greater than 0.20), and Cohen’s *d* for mean comparisons (interpreted as mild to moderate too large for values higher than 0.50; Rea and Parker, 1992). Owing to the number of statistical significance tests, Finner’s correction was applied to control the increase in Type I error (as an alternative method to the classical Bonferroni correction; Finner and Gontscharuk, 2009).

The network analysis was conducted with Gephi version 0.9.2 for Windows,^1^ an open-source package specifically developed for network approaches. Two separate networks were obtained in the study, for the subsamples FA + (*N* = 78, including all patients with a subclinical or positive screening score on the YFAS-2) and FA− (*N* = 131, grouping all patients with a negative screening score on the YFAS-2). The nodes considered for analysis included the sociodemographic profile (sex, chronological age, marital status, and social position index), the psychopathological measures of the study (impulsivity level, DERS, global psychological distress, presence of comorbid mental disorders, suicidal behavior, and duration of BA-related problems), and personality profile (as measured with the seven TCI-R dimensions). These 17 variables measured different areas of the patients, providing a visualization of their global functional capacity, an approach strongly recommended in psychological research (Epskamp et al., 2018b). Undirected edges were defined, and their weight was calculated through partial correlations between all pairs of nodes after controlling for all other nodes in the network. The initial data structure for the network resulted in 17 nodes and 136 potential edges, most of which had very low weights (partial correlations close to 0). To simplify this initially complex structure, only edges that achieved *p* < 0.25 were selected. The rationale for this selection was based on the following (Epskamp et al., 2017): (a) achieving a more parsimonious graph (based on fewer connections between nodes); (b) representing only the most relevant empirical relationships between the variables; and (c) considering that the absence of an edge is not evidence that the association between the nodes is exactly zero. Accordingly, edges with *p*-values greater than 0.25 were considered unlikely to reflect meaningful associations and were not retained. From a methodological perspective, adopting a more liberal threshold increases sensitivity by reducing the risk of prematurely excluding potentially relevant associations, at the cost of lower specificity, whereas more stringent thresholds (e.g., *p* < 0.10) would increase specificity but reduce sensitivity, reflecting a more conservative, confirmatory analytic strategy.

The Gephi system uses the Brandes algorithms for calculating the centrality indices (Brandes, 2001). In this study, eigenvector centrality was interpreted as a measure of the overall relevance of each node within the network. This value is calculated as the weighted sum of the centrality scores of all other nodes connected to a given node; thus, higher values indicate that a node is connected to other highly connected nodes, reflecting its relative importance within the network structure. In simpler terms, nodes with high eigenvector centrality tend to occupy prominent positions in the network because they are linked to other influential nodes.

Closeness centrality was considered a measure of the linkage capacity of each node. It is calculated as the reciprocal of the sum of the shortest paths from a given node to all other nodes in the network. Higher values indicate that a node is, on average, closer to all other nodes in the network. Nodes with the highest closeness centrality are often referred to as “bridging nodes” or simply “bridges.” These nodes act as connectors that reduce the overall distance between nodes, facilitating the spread of information across the network (Valente and Fujimoto, 2010). In practical terms, bridging nodes are well positioned to reflect changes or patterns that are relevant across different parts of the network, making them particularly informative when examining overall network structure.

The Gephi package also allows the identification of empirical clusters of nodes, called “communities” or “modules,” through an automatic process based on the Blondel modularity algorithm (Blondel et al., 2008) and the Latapy clustering coefficient algorithm (Latapy, 2008). Network clustering is particularly useful for analyzing complex structures by partitioning them into communities based on the similarity of their connectivity patterns. Each cluster represents a subgroup of nodes with high connectivity within the group and sparse connections to the rest of the graph.

Other additional global graph measures in this study included the (average) path length (mean of the shortest paths between all pairs of nodes, interpreted as the efficiency of information transport in the network) and the diameter (greatest distance between the two furthest nodes, and therefore the maximum eccentricity of any vertex in the graph) (Brandes, 2001). The density of the graph (defined as the ratio between the number of edges represented in the network and the total number of potential connections) was also calculated.

In this study, FA was treated as a categorical stratification variable, allowing the estimation of distinct network models based on the presence *versus* absence of this condition. Although dimensional scoring approaches may capture subthreshold symptomatology and offer greater statistical power, the categorical approach was selected to enhance clinical interpretability and to align the analyses with established diagnostic frameworks. Given that the primary aim of this study was to visualize and compare patterns of associations using network methodology, a dimensional approach based on continuous YFAS-2 symptom scores would require the estimation of multiple networks across symptom levels, which would not constitute a methodologically meaningful or interpretable analytic strategy in this context.

## 3 Results

### 3.1 Description of the sample

The descriptive statistics for all the variables analyzed in the study are displayed in Table 1. For the total sample, most patients were men (*n* = 135, 64.6%), were unmarried (single: *n* = 141, 67.5%; separated–divorced: *n* = 15, 7.2%), reported secondary education (*n* = 87, 41.6%), were unemployed or inactive (*n* = 129, 61.7%), and pertained to mean-low to low social position indices (*n* = 148, 70.9%). Mean age within the total sample was 35.5 years (SD = 15.5).

**Table 1:** Descriptive statistics for the variables analyzed in the study.

	Total		FA-		FA+		*p*	C-V
	(*n* = 209)		(*n* = 131)		(*n* = 78)			
	*n*	%	*n*	%	*n*	%		
Sex								
Female	74	35.4%	36	27.5%	38	48.7%	0.002*	**0.22** ^**†**^
Male	135	64.6%	95	72.5%	40	51.3%		
Marital status								
Single	141	67.5%	89	67.9%	52	66.7%	0.970	0.02
Married–couple	53	25.4%	33	25.2%	20	25.6%		
Divorced–separated	15	7.2%	9	6.9%	6	7.7%		
Education								
Primary	78	37.3%	48	36.6%	30	38.5%	0.913	0.03
Secondary	87	41.6%	56	42.7%	31	39.7%		
University	44	21.1%	27	20.6%	17	21.8%		
Employment								
Unemployed	129	61.7%	79	60.3%	50	64.1%	0.585	0.04
Employed	80	38.3%	52	39.7%	28	35.9%		
Social								
High	11	5.3%	8	6.1%	3	3.8%	0.912	0.07
Mean-high to high	34	16.3%	21	16.0%	13	16.7%		
Mean	16	7.7%	9	6.9%	7	9.0%		
Mean-low	53	25.4%	32	24.4%	21	26.9%		
Low	95	45.5%	61	46.6%	34	43.6%		

	Mean	SD	Mean	SD	Mean	SD	*p*	|d| C-V
	*n*	%	*n*	%	*n*	%		
Age (years)	35.46	15.50	33.98	16.27	37.94	13.88	0.075	0.26
Comorbid mental disorders								
No	140	67.0%	94	71.8%	46	59.0%	0.047*	0.13
Yes	69	33.0%	37	28.2%	32	41.0%		
Suicidal behavior								
No	182	87.1%	119	90.8%	63	80.8%	0.036*	0.15
Yes	27	12.9%	12	9.2%	15	19.2%		

	Mean	SD	Mean	SD	Mean	SD	*p*	|d|
Age of onset of BA (years)	26.63	13.64	25.85	14.39	27.94	12.24	0.286	0.16
Duration of the BA (years)	6.09	5.78	5.57	5.40	6.96	6.31	0.093	0.24
Impulsivity (UPPS-P total)	135.83	22.93	130.84	20.05	144.22	25.06	0.001*	**0.59** ^**†**^
Emotion (dys)regulation (DERS total)	98.64	19.77	93.06	14.04	108.01	24.10	0.001*	**0.76** ^**†**^
Psychological distress (SCL-90-R GSI)	1.35	0.83	1.06	0.66	1.83	0.87	0.001*	**1.00** ^**†**^
TCI-R novelty seeking	104.25	15.79	103.96	15.30	104.74	16.66	0.730	0.05
TCI-R harm avoidance	109.32	19.76	102.73	17.31	120.38	18.73	0.001*	**0.98** ^**†**^
TCI-R reward dependence	95.64	16.63	94.20	16.78	98.05	16.19	0.105	0.23
TCI-R persistence	97.10	21.43	99.32	21.23	93.37	21.38	0.052	0.28
TCI-R self-directedness	123.46	21.87	128.75	19.55	114.58	22.78	0.001*	**0.67** ^**†**^
TCI-R cooperativeness	130.26	17.46	130.60	17.41	129.69	17.64	0.716	0.05
TCI-R self-transcendence	61.76	15.14	60.52	15.44	63.85	14.46	0.125	0.22

BA, Behavioral addiction. FA−, Food addiction negative screening. FA+, Food addiction subclinical or positive screening. SD, Standard deviation. *Bold indicates statistically significant comparisons (*p* < 0.05 level). ^†^Bold indicates relevant effect sizes (mild to moderate to large).

The comparison between FA− (negative screening on the YFAS-2) *versus* FA + (subclinical or positive screening) showed differences in patients’ sex, psychopathological status, and personality profile. The FA + status was associated with a higher proportion of women, a higher risk of comorbid mental disorders and suicidal behavior, higher impulsivity levels, more DERS, greater psychopathological distress, higher harm avoidance, and lower self-directedness.

### 3.2 Network study

**Figure 1:**
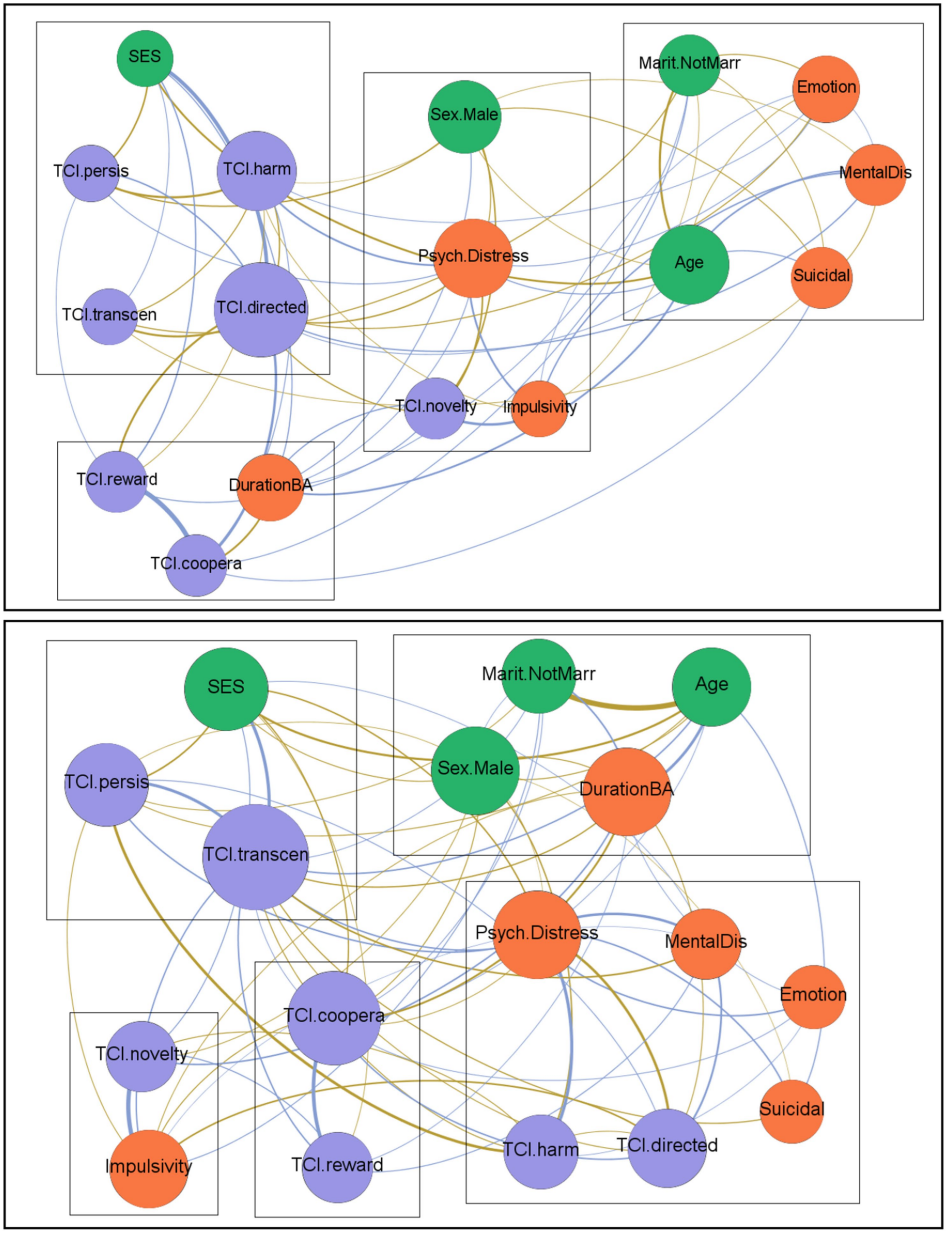
Visualization of the networks. The upper network was obtained for the FA + subsample, and the lower network was obtained for the FA − subsample. Positive edges are represented by blue lines and negative edges by brown–ochre lines. The thicker the edge, the stronger the connection weight. Nodes are plotted in colors depending on the dimension: sociodemographic variables (green), personality factors (purple), and psychopathology measures (orange). Nodes are identified as follows: sex (Sex. Male), chronological age (Age), marital status (Marit. NotMarr), social position index (SES), impulsivity measured with the UPPS-P total (Impulsivity), emotion regulation measured with the DERS total (Emotion), global psychopathological distress measured with the SCL-90-R GSI (Psy. Distress), presence of comorbid mental disorders (MentalDis), suicidal behavior (Suicidal), duration of the BA (DurationBA), and personality profile as measured with the TCI-R (TCI.novelty, TCI.harm, TCI.reward, TCI.persis, TCI.directed, TCI.coopera, TCI.transcen).

Figure 1 provides the visualization of the two networks in the study (Table S1 in the Supplementary material includes the full statistics). In network diagrams, positive edges are represented by blue lines and negative edges by brown–ochre lines; the thicker the edge, the stronger the connection weight. Nodes are plotted in colors depending on the dimension: sociodemographic variables (green), personality factors (purple), and psychopathology measures (orange).

The number of edges defined for the network structure within the FA + subsample was 60, resulting in a density of 0.441. The model showed an average path length of 1.574 and a diameter of 3.0. For the FA− subsample, the network was defined with 68 edges (density of 0.500), achieving an average path length of 1.500 and a diameter of 2.0.

The network structure differed between patients in the FA + *versus* FA− subsamples, as can be visualized in the clustering of the nodes into separate rectangles (Figure 1). For patients with the FA + condition, four communities were identified, with the following composition: (1) social position index, persistence, harm avoidance, self-transcendence and self-directedness; (2) reward dependence, cooperativeness, and duration of the BA; (3) sex, global psychological distress, novelty seeking, and impulsivity; and (4) marital status, age, emotion regulation, comorbid mental disorders, and suicidal behavior. For the FA− subsample, five communities were identified: (1) social position index, persistence, and self-transcendence; (2) novelty seeking and impulsivity; (3) cooperativeness and reward dependence; (4) sex, age, marital status, and duration of the BA; and (5) psychological distress, comorbid mental disorders, emotion regulation, suicidal behavior, harm avoidance, and self-directedness.

**Figure 2:**
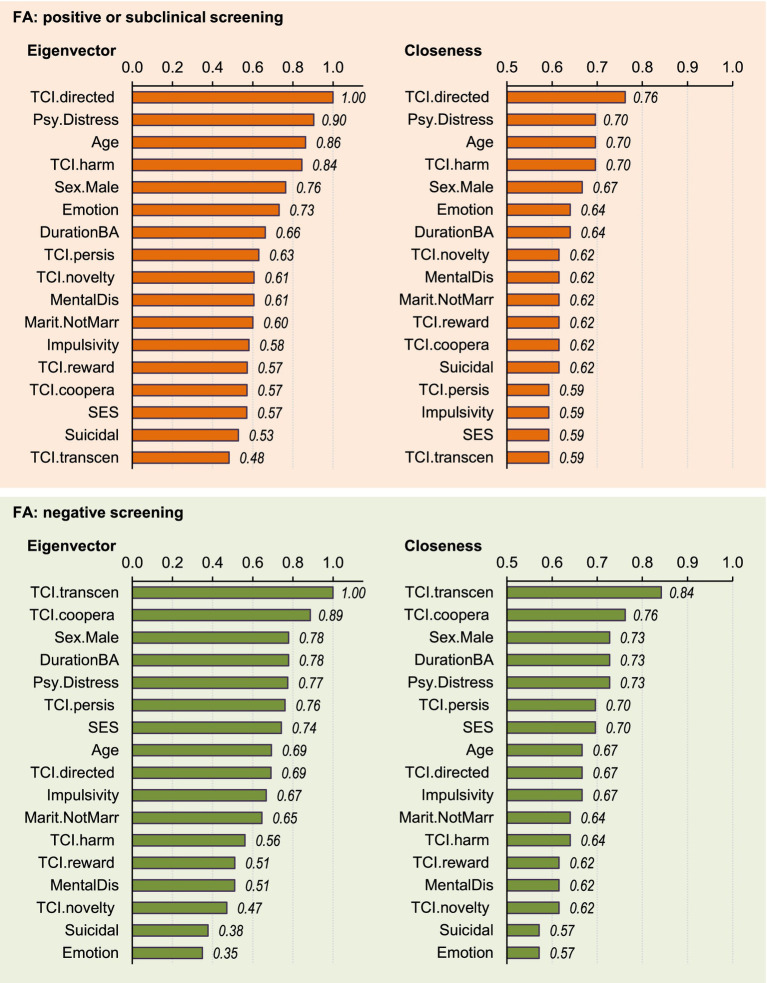
Centrality measures of the nodes: relevance and bridging capacity. Nodes are identified as follows: sex (Sex. Male), chronological age (Age), marital status (Marit. NotMarr), social position index (SES), impulsivity measured with the UPPS-P total (Impulsivity), emotion regulation measured with the DERS total (Emotion), global psychopathological distress measured with the SCL-90-R GSI (Psy. Distress), presence of comorbid mental disorders (MentalDis), suicidal behavior (Suicidal), duration of the BA (DurationBA), and personality profile as measured with the TCI-R (TCI.novelty, TCI.harm, TCI.reward, TCI.persis, TCI.directed, TCI.coopera, TCI.transcen).

The bar charts displayed in Figure 2 show the nodes ordered by eigenvector centrality (importance of each variable in the network) and the closeness centrality (linkage capacity). Within the FA + subsample, the most relevant node was the self-directedness, followed by global psychopathological distress, age, and harm avoidance. Self-directedness was also identified as the bridging node. Within the FA− subsample, the most important node was self-transcendence, which was also identified as the bridge.

**Figure 3:**
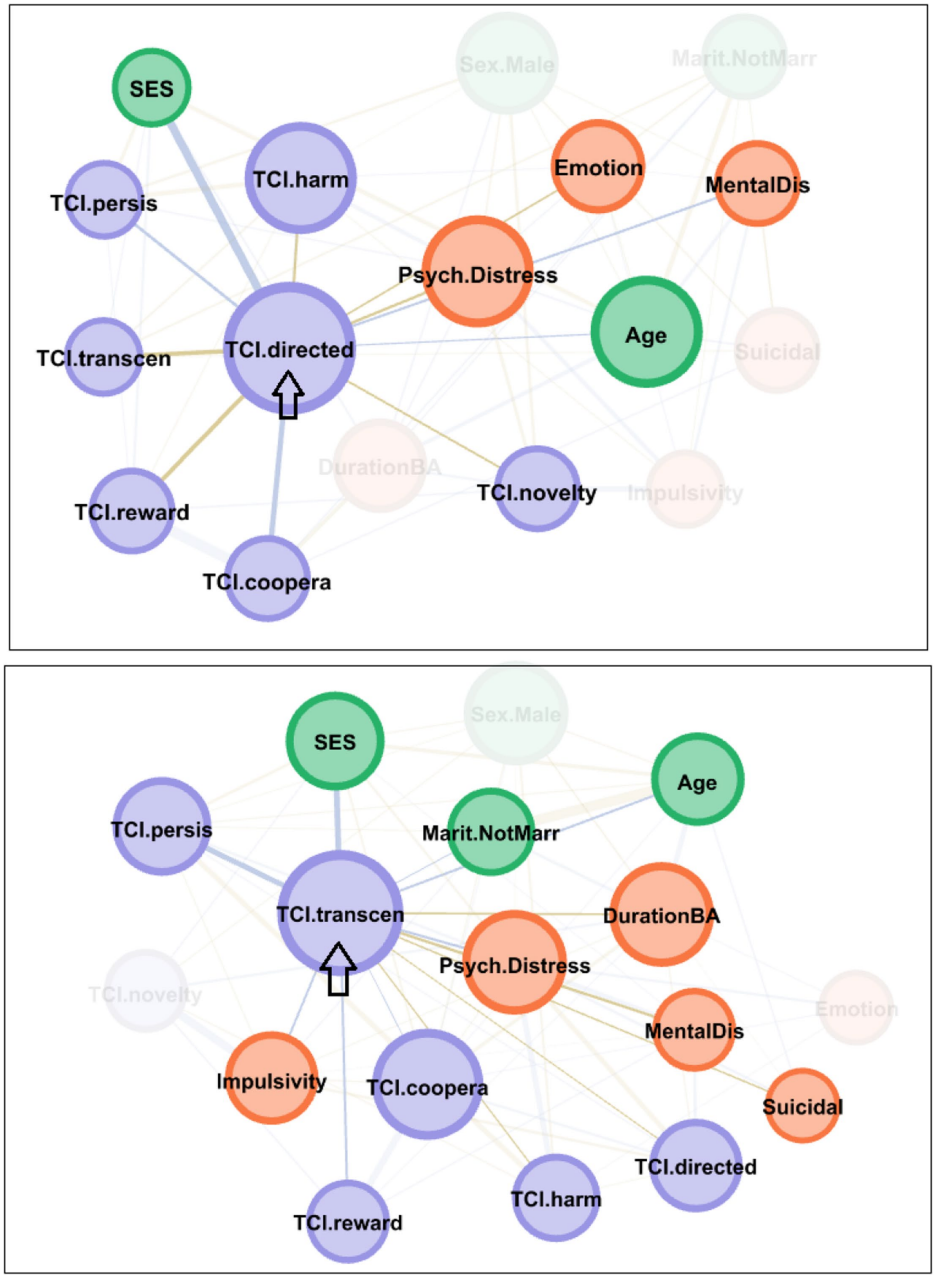
Main linkages for the bridging nodes. The upper network was obtained for the FA + subsample, and the lower network was obtained for the FA − subsample. Positive edges are represented by blue lines and negative edges by brown–ochre lines. The thicker the edge, the stronger the connection weight. Nodes are plotted in colors depending on the dimension: sociodemographic variables (green), personality factors (purple), and psychopathology measures (orange). Nodes are identified as follows: sex (Sex. Male), chronological age (Age), marital status (Marit. NotMarr), social position index (SES), impulsivity measured with the UPPS-P total (Impulsivity), emotion regulation measured with the DERS total (Emotion), global psychopathological distress measured with the SCL-90-R GSI (Psy. Distress), presence of comorbid mental disorders (MentalDis), suicidal behavior (Suicidal), duration of the BA (DurationBA), and personality profile as measured with the TCI-R (TCI.novelty, TCI.harm, TCI.reward, TCI.persis, TCI.directed, TCI.coopera, TCI.transcen).

Figure 3 shows the main linkages for the bridging nodes. The arrow indicates the node that has been selected as “activated,” which highlights its pattern of connections within the network. Nodes with stronger connectivity to the activated node appear more prominently (“activated nodes”), whereas nodes with weaker connections appear faded or less visible (“deactivated nodes”). For the FA + subsample, activation of the self-directedness node had a major impact on most of the other nodes, except for sex, marital status, duration of the BA, impulsivity, and suicidal behavior. For the FA− subsample, the activation of self-transcendence also impacted most of the other nodes, except for sex, novelty seeking, and emotion regulation.

## 4 Discussion

This study applied a network approach to explore the interrelationships between variables measuring the clinical profile of patients seeking treatment for gaming disorder, CBSD, compulsive sexual behavior disorder, and the comorbid presence of multiple BAs. Separate networks were obtained based on the presence or absence of FA. The nodes with the highest centrality indices among patients with FA + were the self-directedness factor, followed by global psychopathological distress, age, and harm avoidance. Self-directedness was also identified as the most relevant and primary bridging node among patients with FA+. The number of communities (clusters of nodes) and their composition differed depending on the comorbid presence of FA.

Regarding the relevance of self-directedness in the networks, previous studies have suggested that specific personality traits may contribute to the onset and maintenance of addiction-related problems because of their impact on decision-making and motivation (Zilberman et al., 2018). For example, low conscientiousness, high neuroticism, and high narcissism have been associated as risk factors for the onset and progression of addictions (Charzyńska et al., 2021). Self-directedness refers to a person’s ability to resolve situations according to a set of objectives and goals (Cloninger, 1993; Cloninger et al., 1997). Individuals with high levels of this personality trait have the capacity to adapt their behaviors to the demands of each situation, as they are particularly motivated to achieve personally chosen goals and values. Other studies have identified low levels of this character dimension as a personality core regardless of mental health diagnosis and chronic course (Fassino et al., 2013a), as well as a predictor of patients’ recovery (de Vries et al., 2021).

In this study, the FA + group registered lower mean levels of self-directedness than FA− patients, and this result suggests that these patients may exhibit greater difficulty in decision-making tasks and may be less successful in achieving long-term goals; therefore, their behavior would be more oriented toward achieving short-term pleasurable experiences. Compared with control samples, lower self-directedness means have also been found in patients with various addictive disorders (Nordin and Nylander, 2007; Milivojevic et al., 2012), including gambling disorder (Nordin and Nylander, 2007) and maladaptive eating behaviors (Wolz et al., 2016; Ouellette et al., 2017).

Low self-directedness also emerged as the bridging node with the highest connectivity with the other nodes, with the exception of sex, marital status, duration of a BA, impulsivity, and suicidal behavior. The high connectivity of this node reflects its central role within the network, as it may contribute to promoting psychopathological distress by propagating symptom activation (Granero et al., 2021a). The activation of this node among patients with FA, characterized by low skills to adapt and self-regulate behaviors, is related to more dysfunctional behaviors and to the search for short-term rewarding behaviors (such as highly palatable foods).

Harm avoidance presents as anticipatory worry and fear of uncertainty, in which responses to unpleasant stimuli, punishment, and non-reward tends to be inhibited (Gutierrez-Zotes et al., 2015), indicating that patients with FA + BA comorbidity tend to show greater difficulty adapting to changes and situations, along with a greater tendency to experience mood swings, tension, apprehension, insecurity, and fears.

Another variable with high centrality in the FA + group was global psychological distress. A high likelihood of comorbid psychopathology has been previously related to the presence and course of BAs (Mallorquí-Bagué et al., 2017; Iyer et al., 2020; Horsager et al., 2025). Additionally, emotional distress is higher in the presence of BAs and FA (Etxandi et al., 2021; Gaspar-Pérez et al., 2025). It has been hypothesized that patients who experience psychological distress could exhibit addictive eating habits and/or high consumption of hyperpalatable foods as a way to cope with stress and negative moods and that FA may play a mediating role between psychological distress and other BAs (Piccinni et al., 2021). In any case, BAs and FA could be considered dysfunctional strategies to alleviate negative mood states (Jiménez-Murcia et al., 2017a). The Interaction of Person-Affect-Cognition-Execution (I-PACE) model is consistent with this proposal. This etiological model explains that the development and maintenance of BAs are based on a dynamic interactive process that includes individual features, emotional responses, cognitive processes, and behavioral expressions. According to this model, the course of BAs may result in several trajectories, which differ significantly depending on changes in symptom severity and the impact of transdiagnostic mechanisms. The model also suggests that in the early stages of BAs (characterized by problematic and risky behaviors), positive feelings, reward, or pleasure are the main driving motivations. In contrast, the negative reinforcement typical of later stages of the BAs (proximal to the initial phases of the disorder) is more driven by compensatory experiences that seek to alleviate negative mood states. In the advanced stages after diagnosis, mood states may be more intense, and individuals might increase compensatory behaviors to attenuate the negative consequences that they perceive. The I-PACE model suggests that both gratification and compensatory experiences are involved in all stages of BAs, but their expression differs depending on the developmental stage of the disorder (Brand et al., 2025). Therefore, both FA and BAs could be associated with gratification (positive reinforcement) and alleviating discomfort (negative reinforcement) (Gaspar-Pérez et al., 2025).

Chronological age also obtained high relevance in the network visualized for the FA + patients. Among this group, the mean age was 37.9 years, younger than the mean reported in other studies with samples characterized by the presence of FA (45–65 years) (Burrows et al., 2018). However, the mean age in our work is similar to the values reported in studies carried out with patients with BAs (Potenza et al., 2019; Severo et al., 2020), except for those analyzing gaming disorder (which reported even younger chronological age, especially in male subsamples) (Chen et al., 2018). Chronological age is, therefore, a variable that should be given close attention, as even at young ages, it may be an indicator of high-risk comorbid profiles. Previous studies have observed that being less than 50 years of age has been suggested as a risk factor associated with psychiatric comorbidity (Löwe et al., 2003). However, in the case of BAs, this threshold should be reviewed, given the common early age of onset of concrete BA types (adolescence and early adulthood), as well as the high likelihood of comorbidity in more severe forms. For example, it has been observed that youth and young adults exhibit a more rapid progression into problematic forms of addictive behaviors than older adult groups (up to twice as fast), with comparable clinical profiles (Hofstedt and Söderpalm Gordh, 2024).

The pattern of linkages between the nodes with high centrality is another interesting result in this work. Within the FA + group, the node measuring self-directedness achieved a high association with the node measuring harm avoidance (a negative relationship was observed, as lower self-directedness was associated with higher harm avoidance). This result could be explained by considering that low self-directedness is related to a lack of decision-making skills, which may lead patients to passively avoid punishment and problems that arise daily, and to perceive less control over their decisions and less motivation to make changes (Cloninger, 1993; Fassino et al., 2013a). These results highlight the central role of personality in FA–BA comorbidity, particularly self-directedness and harm avoidance. Assessing these traits and integrating them into personalized, transdiagnostic interventions may enhance self-regulation, decision-making, and adaptability, potentially improving treatment efficacy and long-term outcomes. Early identification of low self-directedness and high harm avoidance could also help detect individuals at greater risk, supporting interventions that target personality dimensions across psychopathology. Cognitive-behavioral therapy (CBT), widely used for addictive behaviors and maladaptive eating patterns (Ayub et al., 2023; Legendre and Bégin, 2023; Reangsing et al., 2025), provides a suitable framework to address these processes (Adams et al., 2019). However, evidence-based treatments exist mainly for gambling disorder (Kim and Hodgins, 2021), while interventions for FA are limited and their effectiveness remains unclear (Florio et al., 2022). The scarcity of protocols targeting FA–BA comorbidity highlights a significant clinical gap, to which this study contributes by supporting personality traits as relevant therapeutic targets. In addition to standard CBT procedures, integrating complementary therapeutic components may further enhance treatment efficacy, particularly for individuals with pronounced difficulties in emotion regulation and self-directed functioning. Evidence supports the use of mindfulness-based strategies to increase tolerance of distressing internal experiences and foster a non-judgmental stance toward negative affective states (Raugh et al., 2025). These skills are especially relevant for patients exhibiting heightened emotional reactivity or engaging in maladaptive avoidance behaviors (Perczel-Forintos et al., 2025). Incorporating brief mindfulness exercises, such as focused-attention practices, body-scan training, and mindful breathing, can thus promote greater emotional stability during the therapeutic process. Additionally, including targeted emotion-regulation techniques (e.g., emotional identification, cognitive reappraisal, and behavioral activation), along with interventions aimed at strengthening self-esteem and self-efficacy, may further reduce maladaptive cognitive and behavioral patterns (Chin et al., 2024; Southward et al., 2024). Finally, structured modules focusing on planning, problem-solving, and adaptive decision-making can significantly enhance self-directedness, executive functioning, and more deliberate, goal-oriented behavior.

Moreover, the FA + group was characterized by a higher impulsive profile, more difficulties in emotion regulation, a greater risk of comorbid mental disorders and suicidal behavior, and a higher percentage of women (48.7%). Being a woman has been identified as a risk factor for the presence of FA (Burrows et al., 2018; Schulte and Gearhardt, 2018) and for various mental disorders (Horsager et al., 2021). The association between the presence of FA and female sex has also been reported even for those mental disorders where the proportion of men tends to be higher, such as autism spectrum disorders (Horsager et al., 2021) and gambling disorder (Jiménez-Murcia et al., 2017a; Etxandi et al., 2021). Considering diverse BA subtypes, the comorbid presence of these disorders with FA has been associated with a worse psychological state, more DERS, and higher impulsivity (Gaspar-Pérez et al., 2025). This evidence has also been observed in samples of bariatric surgery candidates (Müller et al., 2018).

However, research on the comorbid presence of BAs and FA + remains scarce. Some studies have suggested that FA may be an indicator of complex traumatic antecedents and the intensity of psychiatric comorbidity (Brewerton, 2017). For example, studies of suicidal behavior have shown that these tendencies are frequent among patients with BAs, with gambling disorder showing the highest percentage of suicidal ideation, followed by CBSD, compulsive sexual behavior disorder, and gaming disorder, while the highest prevalence of suicidal attempts is related to compulsive sexual behavior disorder, followed by CBSD. These findings indicate that suicidal behavior is prevalent in BAs (Valenciano-Mendoza et al., 2021). Considering FA, one study reported that this condition is a predictor of NSSI across the lifespan among patients with EDs (Carlson et al., 2018). It has also been found that among women, the presence of FA increases the likelihood of NSSI in both EDs and healthy controls (Carlson et al., 2018). In candidates for bariatric surgery, a significant association has also been found between FA and increased suicidal tendencies (Benzerouk et al., 2018).

In addition, high impulsivity is a construct strongly associated with FA (Gaspar-Pérez et al., 2023), as well as with substance-related addictions and BAs (Zilberman et al., 2018; Seong et al., 2019; Iyer et al., 2020; Granero et al., 2021a). This connection suggests that patients with these clinical conditions exhibit a greater tendency for immediate reinforcement despite long-term losses (Zilberman et al., 2018). In this context, a study using the Iowa Gambling Task (IGT), one of the most widely recognized computerized neurocognitive assessments for decision-making, found that patients with BAs, such as gambling disorder, are more likely to exhibit hypersensitivity to reward rather than to punishment and that high impulsivity is associated with decision-making under risk (Ochoa et al., 2013). Similarly, a meta-analysis found that in both SUDs and BAs, highly impulsive individuals tend to disregard the consequences of their decisions. The findings of this meta-analysis, based on performance in the IGT, also indicated a greater decision-making deficit in individuals with gambling disorder than in those with alcohol use disorder, suggesting that impaired decision-making is more closely associated with the addictive behavior itself than with substance consumption per se (Kovács et al., 2017). In this study, impulsivity levels increased even more with the presence of FA, suggesting that these patients tend to respond impulsively to both negative and positive emotions.

Our results suggest that patients with BA and FA + may constitute a more vulnerable group than those with BA without FA (FA-), with a more complex network structure. Previous research has observed that the comorbidity of multiple addictions is associated with worse psychosocial functioning (Charzyńska et al., 2021).

In addition, within the FA- subsample, the most important node was self-transcendence, which was also identified as the bridging node. Self-transcendence is associated with spiritual experiences, a connection to nature, sensitivity, creativity, and reduced materialism (Cloninger, 1999). However, dysfunctional scores on this dimension in clinical samples may facilitate the isolation of the individuals, leading them to immerse themselves in activities that they perceive as rewarding without the need to interact face to face with others (Cloninger, 1993; Rezaei et al., 2020). This personality trait has been identified as a central node in patients with gambling disorder and gaming disorder (Granero et al., 2021, 2024), leading them to focus their attention on addictive behaviors while ignoring other internal or external stimuli, despite negative consequences.

Finally, our results supported the hypothesis that network sub-structures differ according to the presence or absence of FA. The communities (clusters of nodes) identified within each condition (FA + *vs*. FA-) may suggest differences in the endophenotypes that should be considered in the development of future treatment and prevention plans.

## 5 Limitations and strengths

The results of this study should be interpreted with caution. First, this is a pioneering study exploring the comorbidity between FA and different BAs through network analysis, and the interpretation of the results might be limited because there have been few previous empirical studies. Second, this cross-sectional design does not provide evidence regarding potential causal or directional relationships. Another limitation was the origin of the sample: all participants were recruited from a hospital setting, which limits the generalizability to other clinical or population-based settings. The small sample size for the FA + group was also a limitation, which could affect statistical power, network stability, and robustness. To partly address this issue, the initial network structure was simplified by including only associations with *p* < 0.25, substantially reducing the number of possible edges and facilitating interpretation. Furthermore, another limitation of the study is that other behavioral addictions have a lower prevalence in clinical practice than gambling disorder, which prevented the conduct of individualized network analyses for each BA. Finally, although the comorbid presence of other addictive behaviors, as well as the global psychological distress, was incorporated into the network models, the presence of concurrent specific psychiatric diagnoses was not analyzed. Future studies should analyze the specific networks of patients with individual BAs and comorbid FA to clarify disorder-specific mechanisms and inform the design of targeted intervention strategies focusing on central and bridging nodes. Longitudinal network analyses are also required to explore directional effects, potential causal relationships, and treatment-response networks. This approach would improve understanding of the dynamics of comorbidity and the role of highly connected symptoms, whose modification could influence the broader symptom network. Empirically testing these relationships and targeting central symptoms are particularly relevant for comorbid conditions such as FA and BAs, where they may enhance intervention effectiveness.

Despite these limitations, this study also had several strengths. First, the use of a novel methodology, such as network analysis, enabled the exploration of interrelationships among a wide range of variables measuring the clinical profile of patients with different BAs and comorbid FA. This approach enhances the ability to visualize the structure of interrelationships between variables (including mental symptoms and other features), identify the most central nodes, and detect the existence of modularities.

## 6 Conclusion

This network study provides empirical evidence on the profile of patients with comorbid BA and FA, intending to identify the most relevant associations among a large set of variables, including personality traits, emotional distress, and other measures that contribute to this complex comorbidity. Personality traits were identified as the core nodes in both patients with FA (lower self-directedness and higher harm avoidance) and those without this addictive problem (higher self-transcendence). As personality traits were also identified as the bridging nodes, future intervention programs should consider these variables due to their high connective capacity and potential role as transdiagnostic elements.

The presence or absence of FA influenced the structure of the networks. Previous studies have observed that the presence of this condition is associated with worse psychopathological states, more difficulties in emotional regulation strategies, higher impulsivity, a higher risk of other comorbid mental disorders, and a higher likelihood of suicidal behavior. The results of this study also indicate that the endophenotype observed in patients with BA and FA is more complex than that of patients without FA. This observed complexity is relevant for understanding the clinical presentation and may inform treatment planning. In this context, the network approach represents a highly valuable clinical tool, as it not only deepens theoretical understanding but also provides strong translational potential by identifying central symptoms and key connections that may serve as therapeutic leverage points, thereby informing the design of more targeted and effective interventions for patients with comorbidity between FA and BAs. Future studies should analyze the specific networks of patients with individual BAs and comorbid FA to deepen the understanding of their specific mechanisms and obtain concrete information to design precise intervention plans that include strategies focused on the nodes with the highest relevance and the bridging nodes.

## Data Availability

The raw data supporting the conclusions of this article will be made available by the authors, without undue reservation.

## Footnotes

1 Available at http://gephi.org.

## Glossary

FA: Food Addiction
SUD: Substance use Disorder
BMI: Body Mass Index
PTSD: Post-Traumatic Stress Disorder
ADHD: Deficit Hyperactivity Disorder
NSSI: Non-suicidal Self-injury
EDs: Eating Disorders
BED: Binge Eating Disorder
BN: Bulimia Nervosa
BAs: Behavioral Addictions
CBSD: Compulsive Buying–Shopping Disorder
ICD: Impulse Control Disorder
OCD: Obsessive-Compulsive Disorder
PUI: Problematic Use of the Internet
PSU: Problematic Smartphone Use
DSM: Diagnostic and Statistical Manual of Mental Disorders
YFAS-2: Yale Food Addiction Scale 2.0
PBS: Pathological Buying Screener
UPPS-P: Impulsive Behavior Scale
NU: Negative Urgency
LP: Lack of Perseverance
LPM: Lack of Premeditation
SS: Sensation Seeking
PU: Positive Urgency
DERS: Difficulties in Emotion Regulation Strategies
TCI-R: Temperament and Character Inventory—Revised
SCL-90-R: Symptom Checklist—Revised
GSI: Global severity index
PSDI: Positive symptom distress index
PST: Positive symptom total
χ^2^: Chi-square
(I-PACE) model: Person-Affect-Cognition-Execution
IGT: Iowa Gambling Task
CBT: Cognitive-behavioral therapy
