# Supplementary material for: Food addiction in behavioral addictions: a network approach
Source: Front Psychol. 2026 Feb 25;17:1703292. doi: 10.3389/fpsyg.2026.1703292 (PMC12975927; doi:10.3389/fpsyg.2026.1703292)
Supplement: Supplementary file 1 [file Table_1.docx]

Supplementary Material

***Table S1 (Supplementary)*** *Results of the network within the subsamples of patients with FA+ and FA−*

| Label | Dimension | ID | Closeness  centrality | Harmonic  closeness  centrality | Betweeness  centrality | Authority | HUB | Modularity | Clustering.  coefficient | Number  triangles | Eigenvector  centrality |
| --- | --- | --- | --- | --- | --- | --- | --- | --- | --- | --- | --- |
| *Subsample FA+* |  |  |  |  |  |  |  |  |  |  |  |
| Sex (male) | Sociodemographic | Sex.Male | 0.6667 | 0.7500 | 6.8912 | 0.2679 | 0.2679 | 2 | 0.3929 | 11 | 0.7642 |
| Age (years-old) | Sociodemographic | Age | 0.6957 | 0.7813 | 6.9161 | 0.3020 | 0.3020 | 4 | 0.4444 | 16 | 0.8629 |
| Marital (not married) | Sociodemographic | Marit.NotMarr | 0.6154 | 0.7083 | 5.0833 | 0.2094 | 0.2094 | 4 | 0.3333 | 7 | 0.6004 |
| Social position index | Sociodemographic | SES | 0.5926 | 0.6771 | 3.1912 | 0.1997 | 0.1997 | 1 | 0.4000 | 6 | 0.5706 |
| Impulsivity (UPPS-P total) | Psychological | Impulsivity | 0.5926 | 0.6771 | 2.6719 | 0.2038 | 0.2038 | 2 | 0.4000 | 6 | 0.5808 |
| Emotion (DERS total) | Psychological | Emotion | 0.6400 | 0.7188 | 2.4190 | 0.2563 | 0.2563 | 4 | 0.5714 | 12 | 0.7307 |
| Psych.Distress (SCL-90R GSI) | Psychological | Psy.Distress | 0.6957 | 0.7813 | 5.4494 | 0.3174 | 0.3174 | 2 | 0.5000 | 18 | 0.9040 |
| Comorbid mental disorders (yes) | Psychological | MentalDis | 0.6154 | 0.6875 | 2.3111 | 0.2120 | 0.2120 | 4 | 0.4667 | 7 | 0.6050 |
| Suicidal behavior | Psychological | Suicidal | 0.6154 | 0.6875 | 3.2193 | 0.1841 | 0.1841 | 4 | 0.4000 | 6 | 0.5283 |
| Duration of BA (years) | Psychological | DurationBA | 0.6400 | 0.7188 | 4.4912 | 0.2316 | 0.2316 | 3 | 0.3810 | 8 | 0.6618 |
| TCI-R novelty seeking | Personality | TCI.novelty | 0.6154 | 0.6875 | 2.7333 | 0.2130 | 0.2130 | 2 | 0.4000 | 6 | 0.6067 |
| TCI-R harm avoidance | Personality | TCI.harm | 0.6957 | 0.7813 | 8.2994 | 0.2961 | 0.2961 | 1 | 0.3889 | 14 | 0.8445 |
| TCI-R reward dependence | Personality | TCI.reward | 0.6154 | 0.6875 | 2.1971 | 0.2005 | 0.2005 | 3 | 0.5333 | 8 | 0.5723 |
| TCI-R persistence | Personality | TCI.persis | 0.5926 | 0.6771 | 1.6944 | 0.2211 | 0.2211 | 1 | 0.6000 | 9 | 0.6299 |
| TCI-R self-directedness | Personality | TCI.directed | 0.7619 | 0.8438 | 13.6383 | 0.3504 | 0.3504 | 1 | 0.3636 | 20 | 1.0000 |
| TCI-R cooperativeness | Personality | TCI.coopera | 0.6154 | 0.6875 | 4.3579 | 0.1995 | 0.1995 | 3 | 0.3333 | 5 | 0.5716 |
| TCI-R self-transcendence | Personality | TCI.transcen | 0.5926 | 0.6563 | 2.4357 | 0.1680 | 0.1680 | 1 | 0.3000 | 3 | 0.4816 |
| *Subsample FA−* |  |  |  |  |  |  |  |  |  |  |  |
| Sex (male) | Sociodemographic | Sex.Male | 0.7273 | 0.8125 | 6.7417 | 0.2773 | 0.2773 | 5 | 0.4667 | 21 | 0.7784 |
| Age (years-old) | Sociodemographic | Age | 0.6667 | 0.7500 | 2.1861 | 0.2475 | 0.2475 | 5 | 0.6429 | 18 | 0.6934 |
| Marital (not married) | Sociodemographic | Marit.NotMarr | 0.6400 | 0.7188 | 0.8028 | 0.2307 | 0.2307 | 5 | 0.7619 | 16 | 0.6461 |
| Social position index | Sociodemographic | SES | 0.6957 | 0.7813 | 4.6694 | 0.2646 | 0.2646 | 1 | 0.5278 | 19 | 0.7426 |
| Impulsivity (UPPS-P total) | Psychological | Impulsivity | 0.6667 | 0.7500 | 3.2583 | 0.2380 | 0.2380 | 2 | 0.5357 | 15 | 0.6674 |
| Emotion (DERS total) | Psychological | Emotion | 0.5714 | 0.6250 | 0.6000 | 0.1243 | 0.1243 | 3 | 0.5000 | 3 | 0.3493 |
| Psych.Distress (SCL-90R GSI) | Psychological | Psy.Distress | 0.7273 | 0.8125 | 8.0694 | 0.2758 | 0.2758 | 3 | 0.4889 | 22 | 0.7746 |
| Comorbid mental disorders (yes) | Psychological | MentalDis | 0.6154 | 0.6875 | 1.3000 | 0.1817 | 0.1817 | 3 | 0.6000 | 9 | 0.5098 |
| Suicidal behavior | Psychological | Suicidal | 0.5714 | 0.6250 | 0.2778 | 0.1345 | 0.1345 | 3 | 0.6667 | 4 | 0.3771 |
| Duration of BA (years) | Psychological | DurationBA | 0.7273 | 0.8125 | 7.0944 | 0.2774 | 0.2774 | 5 | 0.4667 | 21 | 0.7783 |
| TCI-R novelty seeking | Personality | TCI.novelty | 0.6154 | 0.6875 | 2.1667 | 0.1673 | 0.1673 | 2 | 0.4000 | 6 | 0.4702 |
| TCI-R harm avoidance | Personality | TCI.harm | 0.6400 | 0.7188 | 3.1778 | 0.1998 | 0.1998 | 3 | 0.4762 | 10 | 0.5615 |
| TCI-R reward dependence | Personality | TCI.reward | 0.6154 | 0.6875 | 1.3167 | 0.1818 | 0.1818 | 4 | 0.6000 | 9 | 0.5104 |
| TCI-R persistence | Personality | TCI.persis | 0.6957 | 0.7813 | 3.4944 | 0.2716 | 0.2716 | 1 | 0.5556 | 20 | 0.7615 |
| TCI-R self-directedness | Personality | TCI.directed | 0.6667 | 0.7500 | 2.2917 | 0.2461 | 0.2461 | 3 | 0.6071 | 17 | 0.6905 |
| TCI-R cooperativeness | Personality | TCI.coopera | 0.7619 | 0.8438 | 7.5278 | 0.3162 | 0.3162 | 4 | 0.4909 | 27 | 0.8872 |
| TCI-R self-transcendence | Personality | TCI.transcen | 0.8421 | 0.9063 | 13.0250 | 0.3564 | 0.3564 | 1 | 0.4615 | 36 | 1.0000 |
